# Novel Perspectives for the Management of Multilingual and Multialphabetic Heritages through Automatic Knowledge Extraction: The DigitalMaktaba Approach †

**DOI:** 10.3390/s22113995

**Published:** 2022-05-25

**Authors:** Sonia Bergamaschi, Stefania De Nardis, Riccardo Martoglia, Federico Ruozzi, Luca Sala, Matteo Vanzini, Riccardo Amerigo Vigliermo

**Affiliations:** 1 University of Modena and Reggio Emilia, 41125 Modena, Italy; sonia.bergamaschi@unimore.it (S.B.); federico.ruozzi@unimore.it (F.R.); r.a.vigliermo@unimore.it (R.A.V.); 2 mim.fscire, 40125 Bologna, Italy; denardis@fscire.it (S.D.N.); salaluca.info@gmail.com (L.S.); vanzinimatteo.info@gmail.com (M.V.)

**Keywords:** digital libraries, minority languages, humanistic informatics, computer archiving, intercultural communication

## Abstract

The linguistic and social impact of multiculturalism can no longer be neglected in any sector, creating the urgent need of creating systems and procedures for managing and sharing cultural heritages in both supranational and multi-literate contexts. In order to achieve this goal, text sensing appears to be one of the most crucial research areas. The long-term objective of the *DigitalMaktaba* project, born from interdisciplinary collaboration between computer scientists, historians, librarians, engineers and linguists, is to establish procedures for the creation, management and cataloguing of archival heritage in non-Latin alphabets. In this paper, we discuss the currently ongoing design of an innovative workflow and tool in the area of text sensing, for the automatic extraction of knowledge and cataloguing of documents written in non-Latin languages (Arabic, Persian and Azerbaijani). The current prototype leverages different OCR, text processing and information extraction techniques in order to provide both a highly accurate extracted text and rich metadata content (including automatically identified cataloguing metadata), overcoming typical limitations of current state of the art approaches. The initial tests provide promising results. The paper includes a discussion of future steps (e.g., AI-based techniques further leveraging the extracted data/metadata and making the system learn from user feedback) and of the many foreseen advantages of this research, both from a technical and a broader cultural-preservation and sharing point of view.

## 1. Introduction

**Figure 1:**
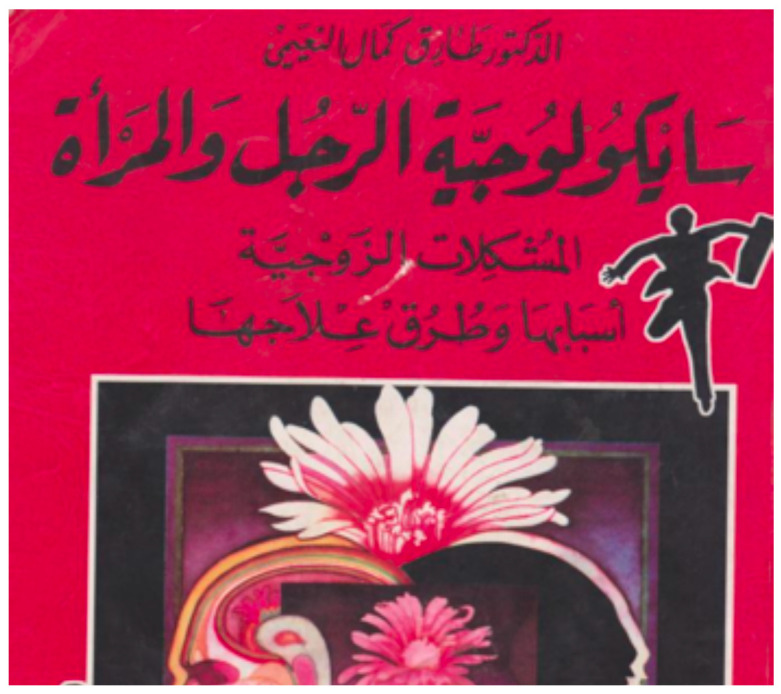
A sample frontispiece with multiple Arab characters.

Since 1700, when the difficulty of establishing a stable system of norms arose, Europe has been studying the management and cataloguing of documentary heritages. Organic codes were devised for catalog compilation in several countries between the 1800s and 1900s, and worldwide agreements were established to create a common system of descriptive cards. The need to manage multimedia content today imposes new and urgent demands: creating systems and procedures for managing and sharing cultural heritages in both supranational and multi-literate contexts. This is the challenging scenario of the recently started *DigitalMaktaba* (in Arabic, the word *maktaba* is derived from the root k-t-b which originates the words: *kitāb* (“book”), *kutub* (“books”), *kātib* (“writer”), *kuttāb* (“writers”, also “Koranic school”) and so on. The prefix *ma-* indicates the place where something is found or carried out; therefore, *maktaba* literally means: the “place where books are found”, “library”) project, born from the collaboration between computer scientists, historians, librarians, engineers and linguists gathered together from the mim.fscire start-up, the University of Modena and Reggio Emilia (UniMoRe) and the Fondazione per le Scienze Religiose (FSCIRE), leader institution of the RESILIENCE European research infrastructure on Religious Studies (ESFRI Roadmap, 2021). The intersection of the knowledge of religious studies, digital humanities, corpus linguistics, educational studies and engineering and computer science guarantees a broad reflection on various aspects related to the design theme: technological, ethical, cultural, social, economic, political and religious. This synergy between academic and extra-academic science-sector skills and varied professional experience is fundamental to effectively address the challenges that a technologically advanced, multicultural and historically rich community, such as the European one, poses in the field of the conservation and enhancement of one’s own cultural heritage. The long-term objective is to establish procedures for the creation, management and cataloguing of librarian and archival heritage in non-Latin alphabets. In particular, the project test case is the large collection of digital books made internally available by the “Giorgio La Pira” library in Palermo, which is a hub of FSCIRE foundation, dedicated to history and doctrines of Islam. Documents such as these pose a number of non-trivial issues in their computer-assisted management, especially optical character recognition (OCR) and knowledge extraction, since their texts are presented in several non-Latin alphabets (in particular, Arabic, Persian and Azerbaijani) and, for each alphabet, in multiple characters, also in a single work (see Figure 1 for a sample).

DigitalMaktaba focuses on innovative solutions in the context of digital libraries, providing several techniques to support and automate many of the tasks (OCR, linguistic-resource linking, metadata extraction, and so on) related to the text sensing/knowledge extraction and cataloguing of the documents in a multi-lingual context. Even if the text sensing/OCR/machine-learning research area is in general very active concerning Latin script documents [1,2], up to now only few projects (e.g., [3,4,5]) have been proposed in the state of the art research for the curation of new and innovative digital libraries in the considered Arabic-script languages; furthermore, most of them require consistent manual work and none of them returns rich information and metadata beyond the extracted text. However, we deem that the linguistic and social impact of multiculturalism can no longer be neglected in any sector. Until a few years ago, only few highly specialized libraries possessed texts in non-Latin alphabets; now, even the smallest ones must adapt acquisitions to the needs of culturally heterogeneous users and are often unable to do so due to the difficulty of managing this data. Hence, the urgency of a global sharing of multicultural heritages.

The present work extends our previous paper [6] in several directions and discusses the currently ongoing design of an innovative workflow and tool for the automatic extraction of knowledge and cataloguing of documents written in non-Latin languages, and in particular for the Arabic, Persian and Azerbaijani languages. The Material and Methods section (Section 3) presents an overview of the tool that is being developed, whose information-extraction pipeline (Section 3.1) smartly combines the output of several techniques that are described in detail, with special emphasis on the text-sensing aspect:

- *Text extraction*, leveraging and combining the output of the best-performing OCR libraries in order to extract text information in a more accurate and uniform way than current proposals;
- *Metadata* enrichment, enabling to automatically capture useful metadata: (a) *syntactic metadata*, including text-regions information (improved w.r.t. the state of the art approaches by means of a newly proposed text-region numbering and merging approach), identified language(s) and character(s), text size and position on page, and the self-assessed quality of extraction through an ad-hoc metric; (b) *linguistic metadata*, including links to external linguistic resources providing useful information such as word definitions for further (semantic) processing; and (c) *cataloguing metadata*, through a novel approach for automatic title and author identification in a frontispiece.

Besides information extraction, which is our current focus, we also take a look (Section 3.2), for the first time, at the data-management foundations enabling convenient and efficient access to the stored data and simple data exchange. Results (discussed in Section 4) include a look at the user interface and overall functionalities of the current prototype incorporating the above-described techniques, and several preliminary evaluation tests. The tests, performed on a subset of our use case dataset provide promising results on the effectiveness of the text, text-regions and cataloguing-metadata extraction, also w.r.t. the state of the art techniques. Generally speaking, the tool already overcomes typical limitations of current proposals, including uneven performance/limited support for different languages/characters, difficulties in automating batch extraction and very limited additional metadata availability.

The discussed techniques and their rich metadata output will be the groundwork for the complete semi-automated cataloguing system we are aiming to obtain, whose future steps, including intelligent and AI-based techniques providing even greater assistance to the librarian and incremental learning with system use, are discussed in Section 4.3. The paper is complemented by a detailed discussion of the state of the art research (Section 2). Finally, Section 5 concludes the paper by detailing some of the many foreseen advantages of this research, both from a technical and broader cultural point of view. In short, we hope this research will ultimately help in preserving and conserving culture, a crucial task, especially in this particular and interesting scenario, and to facilitate the future consultation and sharing of knowledge, thus encouraging the inclusiveness of the European community and beyond.

## 2. Related Works

In this section, we discuss related works by specifically focusing on projects that have been proposed for the curation of digital libraries in Arabic-script languages (Section 2.1). We also specifically examine what is available on the text sensing/extraction front, always in Arabic script (Section 2.2). We conclude the section by comparing the features of the DigitalMaktaba proposal to existing state of the art techniques, specifically identifying the innovative aspects (Section 2.3).

### 2.1. Projects and Proposals for the Curation of Digital Libraries in Arabic-Script Languages

From an academic point of view, even though the information-retrieval and text-extraction/sensing fields on Arabic scripts have made huge strides in the last decades, there have been not many projects aimed at exploiting them for the curation of new and innovative digital libraries. In 2009 the Alexandria library announced the creation of the Arabic Digital Library as a part of the DAR project (Digital Assests Repository), with text-extraction tools for Arabic-language characters implemented with a high accuracy, despite being designed only for extracting short information in the text [7]. In addition, worth mentioning here are more recent projects concerning the digitization and the building of Arabic and Persian texts corpora. The first example is represented by the Open Islamicate Text Initiative (OpenITI) [3], which is a multi-institutional effort to construct the first machine-actionable scholarly corpus of premodern Islamicate texts. Led by researchers at the Aga Khan University International (AKU), University of Vienna/Leipzig University (LU), and the Roshan Institute for Persian Studies at the University of Maryland, OpenITI contains almost exclusively Arabic texts, which were put together into a corpus within the OpenArabic project, which was developed first at Tufts University in the frame of the Perseus Project [8] and then at Leipzig University. The main goal of OpenArabic is to build a machine-actionable corpus of premodern texts in Arabic collected from open-access online libraries such as Shamela [9] and the Shiaonline library [10]. From this important partnership, two other interesting projects have been developed: KITAB [4] at the AKU and the Persian Digital library (PDL) at the Roshan Institute for Persian Studies [5]. The first one provides a toolbox and a forum for discussions about Arabic texts and its main goal is to research relationships between Arabic texts and discover the inter-textual system laying underneath the Arabic rich textual tradition. The PDL project is part of the larger Open ITI project and is focused primarily on the construction of a scholarly verified and machine-actionable corpus. PDL has already created an open-access corpus of more than 60,000 Persian poems collected from the Ganjoor site [11] and then integrated with a lemmatizer [12] and a digital version of the Steingass persian dictionary [13]. Another similar project is Arabic Collections Online (ACO), another multi-institutional project between NYU, Princeton, Cornell, and the American University of Cairo and Beirut in collaboration with the UAE National Archives and the Qatar National Library (QNL). It provides a publicly available digital library of Arabic language content. ACO currently provides digital access to 17,262 volumes across 10,148 subjects drawn from rich Arabic collections of distinguished research libraries [14]. It aims to digitize, preserve, and provide free open access to a wide variety of Arabic language books in subjects such as literature, philosophy, law, religion, and more. Although of a different kind, we would like to mention a few other important projects focusing on the digitization of Arabic and Persian manuscripts that involve handwritten-text recognition (HTR), such as The British Library projects [15,16] with the partnership of the Qatar National Library (Qatar Digital Library) [17] and the Iran Heritage foundation [18].

DigitalMaktaba has a number of significant differences and innovative aspects w.r.t. all the above mentioned approaches; these will be discussed in Section 2.3.

### 2.2. Text Sensing/Extraction/OCR in Arabic-Script Languages

Talking more specifically about text sensing and OCR, one of the areas where the first steps in DigitalMaktaba are being performed, we can distinguish between research projects and publicly available tools. From a research perspective, Arabic-script OCR is not an easy topic, since many issues have to be dealt with, including character skewing, the noisy structure of the titles and the presence of diacritical marks (vowels) mixing with diacritical dots. Studies on hidden Markov models (HMM) such as al-Muhtasib [19] have given good results on character variation. Obaid [20] proposed a segmentation-free approach for the recognition of *naskh*, derived from the verb *nasakha* “to transcribe, to copy, (to abrogate)”, one of the most popoluar forms of Arabic script: now, more Qurans are written in *naskh* than in all other scripts combined. Popular for writing books because of its legibility and adapted for printing, it is still the most common font in printed Arabic. The model is extensible, robust, and adaptive to character variation and to text degradation. The use of symbolic AI combined with algorithms (such as the C4.5 algorithm) has shown high tolerance to noisy documents with a high training speed [21]. In more recent times, contour-based systems for character recognition have been proposed. As shown in the study of Mohammad [22], the systems demonstrate robustness to noise resulting in high average recognition accuracy. Other works have targeted the difficulties posed by Arabic or Persian manuscripts when disentangling overlapped characters that cause diacritic points to nudge forward (right to left) their original position, creating recognition errors or failure. Many attempts have been made to provide useful algorithms able to recognize the slanting and overlapping script typical of the Arabic handwritten script (in particular *nasta’līq*) [23]. Different typologies of neural networks (NN) have been indagated, such as the simple artificial neural network (ANN) [24], bidimensional long-short memory (BLSTM) and recurrent neural network (RNN), sometimes implemented with some HMM [25]. In addition, different ML techniques have been implemented, such as K-means or K-nearest neighbour (KNN), in order to cluster diacritical dots and segment different characters in a proper way. Persian-manuscript recognition has also been an active field of studies. Early in 1997, Dehgan and Faez extracted images utilizing Zernike moments, pseudo-Zernike and Legendre moments [26]. By using an ART2 neural network they obtained very good results. Mowlaei developed a recognition system of Persian digits and characters by using Haar wavelet to extract features and then insert them into an NN [27]. A different approach is represented by fuzzy logic, particularly indicated in ambiguous contexts. Linguistic fuzzy models have demonstrated robustness to Persian script manuscripts variations [28]. More recently, RNN and Deep NN has been introduced along with new segmentation techniques [29] or architectures such as DensNet and Xception [30].

While the above works are certainly interesting, they often do not offer publicly available OCR tools. Therefore, we will now focus specifically on publicly available OCR libraries supporting the required languages. Among the free and open source ones, there are systems such as Tesseract (available online: https://github.com/tesseract-ocr/tesseract (accessed on 4 February 2022)), EasyOCR (available online: https://github.com/JaidedAI/EasyOCR (accessed on 4 February 2022)), GoogleDocs (available online: https://docs.google.com (accessed on 4 February 2022)) and Capture2Text (available online: http://capture2text.sourceforge.net/ (accessed on 4 February 2022)). While certainly a good starting point, these systems have a number of drawbacks that will be discussed in Section 2.3. Regarding metadata extraction, the most notable multilingual resources supporting the considered languages are the Open Multilingual WordNet thesauri (available online: http://compling.hss.ntu.edu.sg/omw/ (accessed on 9 February 2022)), including Arabic and Persian WordNet (see Section 3.1 for more details).

### 2.3. Comparison and Discussion of Innovative Aspects of DigitalMaktaba w.r.t. State of the Art Techniques

Let us now consider the specific contributions of our proposal w.r.t. state of the art techniques, discussing their innovative aspects.

**Overall workflow and tool aim and context**. As seen in Section 2.1, not so many projects have been proposed in this context; in any case, all the projects that we have mentioned target only a part of the languages considered in DigitalMaktaba and aim at the pure digitization of a (smaller) library of books, often with consistent manual work. To give just a brief example, the Italian National Librarian System (SBN) does not provide the opportunity to insert metadata in non-Latin alphabets, thus relying heavily on ineffective transliteration systems, which seems to be in contrast to the adjustments that other countries are preparing and to the standards dictated by the International Standard Bibliographic Description (ISBD). Instead, DigitalMaktaba includes:

- *Multiple languages*: the presented innovative workflow and tool works in the Arabic, Persian and Azerbaijani languages, which have not been considered together in other works;
- *A larger size*: the project is aimed at the creation of a very large digital library (300,000+ books, much more than other projects, which are aimed at thousands of books at most), thanks also to the innovative automation features which are not present in the discussed other projects (see below);
- *Non-Latin alphabet metadata*: the automatically extracted metadata, besides being very rich (see points below), contribute to the creation of a digital library integrated with SBN without relying on transliteration (in contrast to the cited state of the art projects).

**Text-extraction approach**. Regarding text extraction, we have seen in Section 2.2 that, even if some approaches are available in the literature for the considered languages, they are very specific since they do not target all the languages involved in DigitalMaktaba, and, most importantly, they are not publicly available and therefore impossible to be experimentally compared. Concerning the discussed publicly available libraries, considered alone they do not always offer consistent and high-quality results on all the required languages; moreover, many require manual work (batch process is not always possible). Therefore, the novel combined approach we propose in DigitalMaktaba exceeds the scope of the best-performing free libraries and combines/enriches their features (see Section 3.1) in order to obtain a completely automatic system producing high-quality outputs:

- *Better effectiveness*: thanks to the proposed text and text-regions extraction approaches, the achieved effectiveness is better than the state of the art techniques (see tests in Section 4);
- *Across all considered languages*: in contrast to the discussed OCR libraries, all the considered languages are automatically identified without manual work and supported without uneven performance issues.

**Metadata-extraction approach**. Automatic metadata extraction is a unique feature w.r.t. the approaches discussed in Section 2.1 and Section 2.2, which are aimed at pure text extraction and (possibly) manual metadata entering. Instead, DigitalMaktaba offers:

- *Rich metadata*: syntactic, linguistic and cataloguing metadata are extracted and stored for each new processed document;
- *Automated extraction*: the metadata extraction is a fully automated process, including the identification of title and authors, which in state of the art projects (Section 2.1) is performed as a time-consuming manual activity.

**Proposed tool: batch automation, data management and UI**. Further innovative aspects are the following (we are not aware of similar features in the discussed works):

- *Flexible data management*: the system can, at any time, access the data stored in a standard DBMS and convert the bibliographic information toward most desired outputs;
- *Less manual work*: the extraction pipeline and associated UI features help users in performing less manual work, fully automating batch text and metadata extraction (for instance, the discussed OCR libraries do not support this when working with multiple languages).

## 3. Materials and Methods

**Figure 2:**
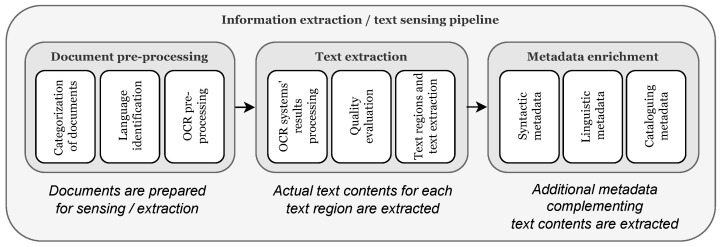
The information-extraction/text-sensing pipeline of the proposed approach: first, documents are pre-processed in order to identify their language and submit them to the available OCR engines (left box); then, text extraction is performed, where OCR raw data is processed and evaluated and the text-region extraction, renumbering, merging and fusion approaches are performed (center box); finally, syntactic, linguistic and cataloguing metadata is sensed (right box). Detailed descriptions of the different phases are available in Section 3.1.

The information-extraction/text-sensing process we propose is depicted in Figure 2 and is divided into three steps, for which we will now give an overview: document preprocessing, text extraction and metadata extraction. Even if the current phase of the project is particularly focused on title pages elaboration, the described approach is sufficiently general for any kind of documents/page; in particular, it is devised so as to provide, for each processed page, information about the identified text regions, the contained text in the best-possible quality and a number of associated metadata.

See Section 3.1 for more details on the processing steps.

**Document preprocessing.** In the first step the documents are classified into *digitized* or *non-digitized* ones. As non-digitized documents do not provide editable text, OCR approaches must be used in order to extract image content (for most-complex case, on which we focus in this paper; for digitized documents, the text is directly extracted and processing goes on to the subsequent steps). To enable effective OCR processing, but also successive metadata extraction, it is necessary to detect in advance the language(s) of the text; in contrast to many state-of-the-art systems, this process is completely automated, then the document is processed by means of several OCR engines, returning a preliminary output which will be processed and merged in the subsequent step.

**Text extraction.** In the second step, the raw output of the OCR engines is analyzed, elaborated and smartly merged in order to extract: (a) for each document page, the different text regions present in it (for instance, a large central text region containing the document title, and so on, a feature that is crucial for automatic cataloguing); (b) for each text region, the contained text with the best-possible quality. The above points require to solve a number of technical issues, including *identification and linking of the different text regions* among the output of the different systems (for (a)) and definition/exploitation of a quality-evaluation metric enabling the *choice/merge of the best text output* (for (b)) (see Section 3.1).

**Metadata enrichment.** Eventually, the output is enriched with additional metadata information, going beyond typical state-of-the-art tools: (i) *syntactic metadata*, i.e., text-regions information, identified language(s) and character(s), text size and position on page, and self-assessed quality of extraction; (ii) *linguistic metadata*, i.e., links to external linguistic resources; and (iii) *cataloguing metadata*, i.e., automatically extracted author and title information (see Section 3.1 for details on their extraction).

### 3.1. Text-Sensing Aspects: Information Extraction

We will now provide more details of the techniques used in the information-extraction steps and the implementation choices behind them. In order to better understand their rationale, we will first of all discuss the preliminary exploratory analyses that were performed on the OCR systems identified in the state-of-the-art approaches and are to be exploited in the processing.

**Analysis and selection of OCR libraries.** Evaluating the best state-of-the-art libraries (and their strengths/weaknesses) on which to base document processing was crucial to define pipeline implementation. In order to reach this aim, we selected a subset of 100 sample documents from the La Pira digital archive, chosen so as to be representative of different languages and characters involved (we will also exploit this subset in the preliminary evaluation of the system compared to state of the art, discussed in Section 4.2). Then, we manually applied several available OCR libraries (including the ones cited in the related work discussion) to test their features and quality. The first filter that allowed us to discard some libraries was the supported language: we eliminated from the choice the libraries that do not support the languages of our interest. Furthermore, we decided to focus on open-source systems, which allowed us to discard many other items from the list. Other tools were discarded as academic projects still under development or carried out at an amateur level that did not seem to suit our purpose. In the end, we selected three libraries: *GoogleDocs* (and in particular its OCR features when importing documents), *EasyOCR* and *Tesseract*.

For testing text-extraction effectiveness, we defined an ad-hoc evaluation framework by taking into account: (a) the quality of the output (*oq*, range [0–2]) as quantified by linguistic experts; (b) the quality of the input (*iq*, range [0–2], taking into account the document scan quality/resolution). Since typical OCR evaluations (including accuracy) are not suited to the above requirements, we defined two ad-hoc *quality metrics* that, from two different points of view, depend on the quality of the documents and also have a strong dependence on expert feedback:

- *qdiff* (range [−2, 2]), expressing whether the output is in line (0), superior (positive values) or inferior (negative values) to the input quality;
- *qscore* (range [1, 5]), expressing the quality of the result of the OCR system given the input quality.

The specific definitions are the following:

(1) $$\mathrm{qscore}=\left\{\begin{array}{cc} 5-((2-oq)*(iq+1)) & \mathrm{if}\;oq\neq 0,\\ 1 & \;\mathrm{otherwise}, \end{array}\right.$$

(2) qdiff=oq−iq

While Equation (2) is quite straightforward, the idea behind Equation (1) is to subtract from the best score a penalization that is the more pronounced the higher the input quality and the lower the obtained output quality. The performed tests (whose numerical results will be summarized in Section 4.2 in comparison with our proposal) highlighted several critical issues in available OCR libraries, with each one having its strengths and weaknesses. On one hand, Tesseract and EasyOCR are capable of extracting a few portions of text with medium quality, and they are among the few to return some metadata (limited to the position of the text in the original image, even if not very precise for Tesseract); on the other hand, they require manual specification of the language before processing. GoogleDocs provides automatic language identification and better output quality; however, at the same time, its output is devoid of metadata. The overall processing pipeline combines such libraries in a new and more comprehensive approach, satisfying our goals for a rich and high-quality output without the need of manual intervention.

**Combined approach, language identification.** On the basis of the technical strengths and weaknesses of the various libraries, specific choices were made to make them work together in an automated way. As to language identification, the documents are first processed with GoogleDocs, whose output is used to obtain the language via GoogleTranslate, then this information is passed to EasyOCR and Tesseract for further processing.

**Text (and text-regions) extraction.** As to text region identification and text extraction, we devised a way to exploit both GoogleDocs (in many cases superior) OCR quality and the other libraries richer output (including text position on the page): for each page, (i) the page is processed in EasyOCR, Tesseract and GoogleDocs in parallel; (ii) from the libraries providing approximate text-region metadata (specifically, EasyOCR, since Tesseract metadata are not sufficiently precise), text-region information is extracted; (iii) text regions are renumbered and merged by means of ad-hoc techniques; (iv) each of the identified regions is “linked” to the text output from the different libraries (including those not supporting region identification, i.e., GoogleDocs); and (v) the best output for each of the regions is selected.

Point (iii) will be described in detail in the following subsection. In order to perform point (iv), the text for each of the regions (from EasyOCR) is compared to (parts of) the raw text obtained from GoogleDocs and Tesseract by means of the edit distance metric: in this way, each sentence (or group of sentences) can be associated to the belonging region. As to point (v), automatic quality evaluation is performed by means of a simple metric wcount defined with the aid of the external linguistic resources. Furthermore, wcount simply corresponds to the count of existing words present within the results of the considered multilingual corpora (Open Multilingual WordNet and others as described in the linguistic metadata description); for each region, the output having the higher wcount is selected.

**Figure 3:**
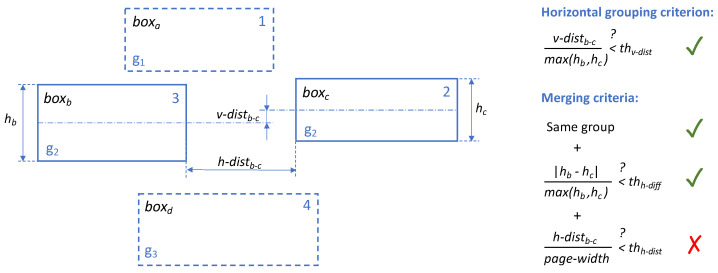
Visual example for horizontal box grouping and merging criteria: $box_{b}$ and $box_{c}$ are grouped into the same horizontal group ($g_{2}$) but are not merged. Groups are shown on the bottom left corner of each box. The resulting box numbering is shown on the top right corner of each box.

**Text (and text-regions) extraction: text-region renumbering and merging.** Text regions (which we will now call boxes, for simplicity) are crucial to the subsequent processing and user-interaction steps; in particular, it is essential to: (a) have them numbered in a way that reflects the logical flow of information; (b) avoid excessive fragmentation (e.g., multiple boxes for information that has to be considered as one piece of text). Unfortunately, due both to the specific complexities given by the considered languages and the often suboptimal quality of the available document images, even the raw output of OCR libraries most suited to box metadata extraction (in our case, EasyOCR) does not meet the above requirements, for instance, fragmenting text into too many boxes and not correctly ordering them following the Arabic right-to-left convention. For this reason, we devised a text-region renumbering and merging phase that proceeds following these steps (see also Abbreviation part for an overview of the used abbreviations):

- **Horizontal grouping:** first of all, the boxes of a page are grouped into horizontal groups following a horizontal grouping criterion (see Figure 3): two boxes will belong to the same group if the vertical distance *v-dist* between their medians w.r.t. the height of the tallest box does not exceed a given threshold *th_v-dist_*;
- **Merging:** inside each group of boxes are boxes satisfying the merging criteria (i.e., relative height difference below a given threshold *th_h-diff_* and horizontal distance relative to page width below a given threshold *th_h-dist_*, as depicted in Figure 3);
- **(Re)numbering:** the resulting boxes are renumbered from top to bottom (different groups) and from right to left (inside each group): Figure 3 shows the resulting numbers on the top right corner of each box.

All thresholds are expressed as a ratio between 0 and 1. As we will see from the tests (Section 4.2), this process enables us to obtain text-region information that is much closer to the desired one (the tests will also discuss how we derive the three best-performing threshold values).

**Metadata-enrichment overview.** As discussed in the pipeline overview, besides text, different metadata are added to the output; let us now detail the different processing phases where each of them comes from. The language of the document is the one extracted from the pre-processing phase; text-region information comes from the text-region extraction/renumbering/merging described in the previous section; text size is not directly available from the OCR libraries output; however, it is extracted by analyzing font sizes in GoogleDocs raw output; quality metadata is the wcount metric corresponding to the best selected output; linguistic metadata is extracted for the document words by searching each of them in the multilingual corpora (this provides additional information including word definitions and synonyms, see next section for more details). Linguistic metadata also enable various methods for the extraction of further cataloguing metadata (automatic identification of title and authors, see next sections) and will as well support future automatic cataloguing tasks (e.g., automatic identification of document topics and categories through semantic processing on linguistic metadata).

**Metadata enrichment: linguistic metadata**. Searching for linguistic information in the languages covered by the project (Arabic, Persian, and Azerbaijani) is certainly a complex task, as we also underlined in the related works: for instance, the Arabic language has many more words and variations, including vocalized and unvocalized, than languages deriving from Latin such as English. To date, there are no open-source linguistic resources providing a coverage level at least comparable to those available for the English language. In order to partially overcome this issue, we decided not to base our tool on a single resource but to exploit a pool of them. After evaluating them in terms of linguistic features and size (i.e., number of words), we designed linguistic-metadata extraction techniques jointly exploiting:

- *Open Multilingual WordNet* (http://compling.hss.ntu.edu.sg/omw/(accessed on 10 March 2022)) providing word lookup for the three different languages (only unvocalized words, in the case of Arabic) and access to extended semantic information (including word definitions). Arabic and Persian/Azerbaijani Wordnet contain 17,785 and 17,560 synsets, respectively;
- *Arramooz* (https://github.com/linuxscout/arramooz (accessed on 10 March 2022)) an open-source Arabic dictionary for morphological analysis providing unvocalized word lookup and definitions for the Arabic language. It contains 50,000 words;
- *Tashaphyne* (https://pypi.org/project/Tashaphyne (accessed on 10 March 2022)), a light stemmer that is used as a devocalizer in order to extend the Arabic coverage of the two previously described resources.

The joint exploitation of the above resources enables us to enhance the overall linguistic coverage: for instance, for the Arabic language, among 458 “test” terms (229 in unvocalized and 229 in vocalized form), we were able to obtain an overall coverage of 73%, compared, for instance, to less than 10% and 36% for Wordnet and Arramooz used alone.

**Metadata enrichment: cataloguing metadata (title/author identification)**. One of the most important but time-consuming activities for cataloguing a new document is to manually insert (or select among the OCR output text) its title, authors and other information. Currently available tools (including those discussed in Section 2) do not propose ways to automate/support this process. The tool we propose aims to exploit the extracted metadata (including box size and position, and linguistic metadata) and text in order to automatically suggest to the librarian the text regions that most likely contain specific fields. At the time of writing, we have designed and tested some preliminary but promising (see Section 4) strategies for identifying the text regions (boxes) containing title and authors in a frontispiece:

- *DIM method*: boxes are sorted on vertical dimension, then the first box in the ranking is suggested as title, the second one as author(s) (following the intuition that the largest texts on a frontispiece are typically the title and the authors’ names, in this order);
- *RES method*: external linguistic resources (including a list of names and surnames in the different languages) are searched and boxes are sorted on the basis of the percentage of found words and names (for identifying title and author, respectively);
- *WGH method*: method combining the contributions of the previous methods (a linear combination ranking fusion technique [31] is exploited in order to produce final rankings for both title and authors).

In the future, we plan to extend these methods and combine them with machine-learning techniques in order to learn from system usage (see also next section).

### 3.2. Data Management

While we are currently most focused on the information extraction techniques which will be key to the effectiveness of our proposal, work is also already undergoing on some of the subsequent steps that will lead to a complete and usable cataloguing tool. In this section, we will specifically discuss the data-management foundations, while a look at the user interface and functionalities we are currently considering on our preliminary prototype implementation will be given in Section 4.1.

The extracted data and metadata are stored on a DBMS in order to guarantee good efficiency levels for both data insertion/update and querying in a typical usage scenario. Currently, our database design is focused on relational DBMSs; in the future, we will also consider extending this design to possibly exploit specific big-data-management techniques and tools for even larger workloads.

**Figure 4:**
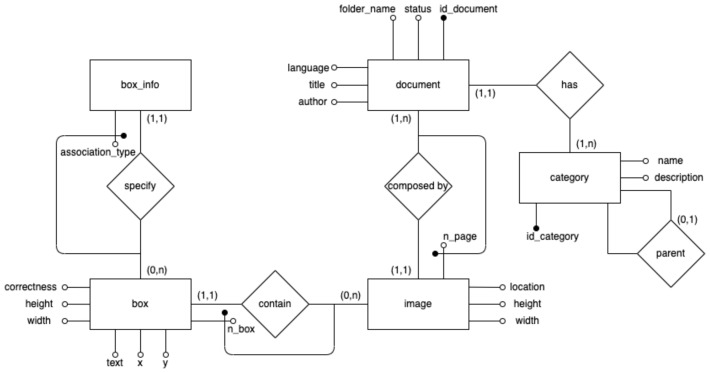
Data management: entity-relationship diagram for database conceptual schema.

Figure 4 shows the entity-relationship schema for our database. The database is designed to easily store and retrieve the document data and metadata whose extraction we described in Section 3.1, along with the definitive catalogue data that the user will insert while using the system:

- The *document* entity is at the heart of the schema and stores the catalogued documents data/metadata, including title, author, language and the path of the folder containing the actual document file(s);
- The details about the scanned images of a document are stored in the *Image* entity (each image corresponds to a specific document page), including file location and image dimensions;
- The *category* entity stores information about the specific category (name and description) to which each document belongs. Categories are currently manually selected by the user from a 3-level hierarchy (modelled through a self-association) containing more than 560 entries; however, in the future we plan to provide “smart” techniques based on AI to assist the process by means of relevant suggestions;
- The entities on the left part of the image store useful metadata about both the OCR results and the feedback coming from system use: for each image, the *Box* entity stores the text regions associated to an image (position, dimensions, text, and automatic quality evaluation as described in previous section), while *Box_info* stores the semantic information about the box content (i.e., the fact that the box contains author, title, and other information is stored in the *association_type* property).

The database is designed to support not only standard cataloguing needs but also to store the data that will be key to provide future smart assitance to the user: in particular, the information contained in *Box* and *Box_info* will enable machine-learning techniques that will be able to enhance the system effectiveness through use (e.g., for title/author recognition).

The database is implemented in PostgreSQL; several kinds of indexes enable its fast querying, in particular GIN (generalized inverted indexes) supporting title and author full-text search and b+trees for category lookup. Further advanced search techniques (including fuzzy approximate search) will be developed in order to make searches more efficient and effective w.r.t. typical cataloguing needs.

## 4. Results and Discussion

In this section, we will consider what we have achieved so far both in terms of the resulting cataloguing-tool prototype we are implementing (whose current user interface and functionalities are described in Section 4.1) and of the experimental evaluation of the presented techniques (Section 4.2).

### 4.1. Prototype: User Interface and Functionalities

**Figure 5:**
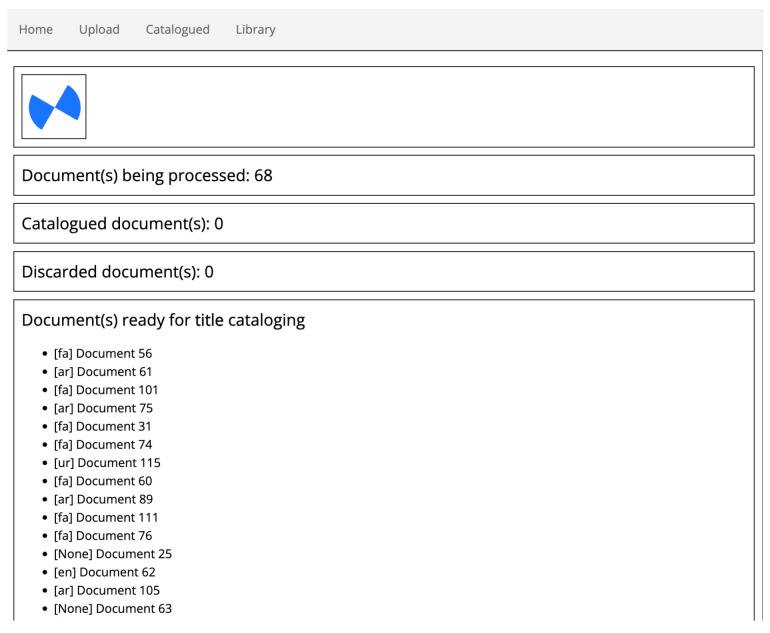
Preliminary resulting cataloguing tool prototype UI: Document preprocessing.

**Figure 6:**
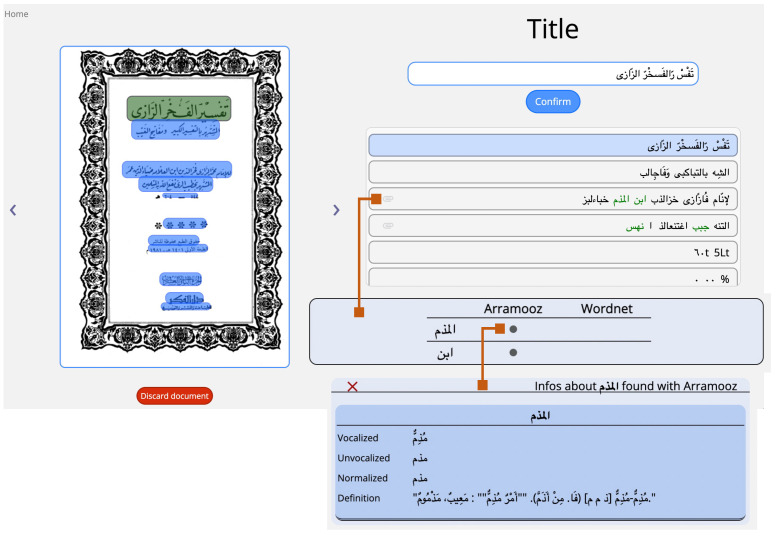
Preliminary resulting cataloguing tool prototype UI: document cataloguing (title window), showing the actual extraction of the title (highlighted in the green box, on the frontispiece displayed on the left). From top to bottom on the left side, the title is hinted before confirming and also selected in blue under the confirm button. Arramoz and Wordnet are the employed linguistic resources, in this case activated (in green) on the word *ibn* “son” and *al-mudhimm* “the one who reprehends, the censor”.

**Figure 7:**
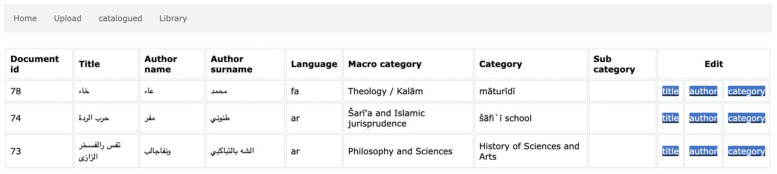
Preliminary resulting cataloguing tool prototype UI: catalogued documents summary. After the semi automatic selection of title, author name (and eventually surname), all selected data are put together in the cataloguing interface in relation to a specific topic and field which represents a category (or sub-category) of the library. Title, author name and surname are shown in the original language (arabic script) as shown here in the figure.

The techniques described in the previous sections have been incorporated in a preliminary application prototype that we are designing. The cataloguing tool is implemented in Python and exploits the Flask framework in order to provide a user-friendly, even if in an initial design phase, user interface. Among the already enabled functionalities are:

- Batch document preprocessing: this allows to preprocess an entire folder of PDF documents. The current implementation exploits multithreading for faster processing, using one OCR thread per page. The UI informs users on the documents being processed and allows them to proceed to document cataloguing for the ones that are ready (see Figure 5);
- Catalogue an already processed document: this guides the user on a series of steps where (s)he is provided with a graphical user interface in order to finalize document properties input (title, author, category, etc.). For instance, the UI for title selection (Figure 6) shows the document frontispiece (on the left) and automatically selects the text region(s) (whose extracted text content is shown on the right) that are most likely to contain the title. The user is able to select different text regions to modify/merge their text (text is concatenated from right to left based on the order in which they are clicked). The tool also visually helps users by showing at a glance the automatically discovered links to the available linguistic resources: the found words are shown in green color and, when the “clip” icon is clicked, a popup displays which words are found in which linguistic resource and, for each of them, related information such as vocalized versions and definitions (lower part of Figure 6). See Section 3.1 for a description of the involved extraction process for text, text regions (including automatic region merge and sorting), and linguistic and cataloguing metadata (including automatic author and title identification);
- Various search functions on the catalogue database, including author and title full-text search and category search (see Section 3.2);
- see a summary of the currently catalogued documents data (Figure 7);
- Other miscellaneous functions: modify already entered data, delete documents from database, restore deleted documents, view catalogued documents.

### 4.2. Experimental Evaluation

In this section, we report on the tests we carried out to perform an initial evaluation of the effectiveness of the approaches we propose (even if the complete tool discussed in the previous section is still in a very early implementation phase). In particular, we will discuss the evaluation of the effectiveness of the text-region renumbering and merging, OCR/text extraction, and of the title and author identification techniques (all described in Section 3.1). All tests are performed on a subset of 100 sample documents from the project library which, thanks to their variety, are representative of the complete collection (both in terms of image quality and linguistic contents). In the future, as the development of the tool continues and as it will be employed for actual librarian and cataloguing work and its database populated, we aim to be able to extend the scope to larger document sets.

**Effectiveness of text-region extraction**. In this first batch of tests, we aimed to evaluate the effectiveness of the text-region renumbering and merging described in Section 3.1. Effectiveness is evaluated on two metrics w.r.t. a gold standard manually determined by experts: *average percentage error*—the percentage of boxes in each document having a wrong number, averaged on the whole document set, and *percentage of documents with box sort error*—the percentage of documents having at least one error in the numbering of their boxes.

**Figure 8:**
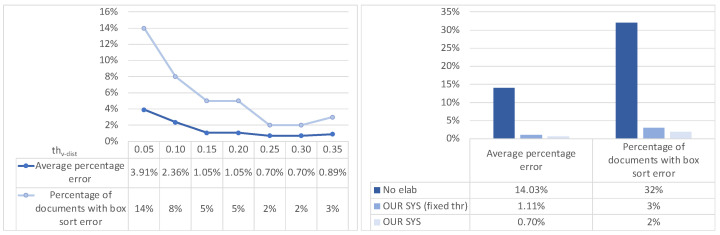
Text-region renumbering tests: effect of vertical-distance threshold *th_v-dist_* (**left**) and error comparison between different approaches (**right**).

Let us first consider text region renumbering. The first test (the left part of Figure 8) shows the effect of moving the vertical distance *th_v-dist_* threshold: as expected, there is a trade off between very low threshold values (which tend to make too-selective horizontal groups) and higher ones (which tend to produce too-inclusive groups).

Note that all threshold “tunings” were performed on a separate tuning dataset of the same size of the main dataset, in order to keep such phases separate from the final evaluation. The trade off is at *th_v-dist_* = 0.25–0.30. This setting enables a very low percentage error of 0.7% for the first metric and 2% for the second one. The right part of Figure 8 compares the final effectiveness achieved by the approach as described in this paper and two baselines (no renumbering, i.e., taking the box region numbers as provided by EasyOCR, and adopting a fixed threshold expressed in pixels instead of the relative one described in this paper): as we can see, the considered metrics drop from 14.03% to 0.7% and from 32% to 2%, respectively.

**Figure 9:**
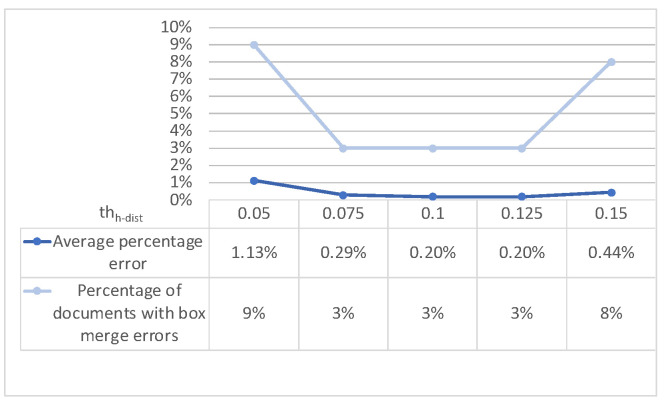
Text-region merging: effect of horizontal-distance threshold *th_h-dist_*.

**Figure 10:**
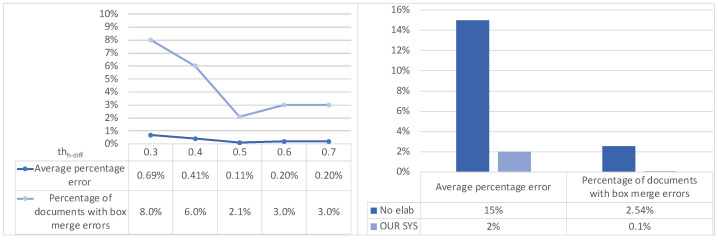
Text-region merging: effect of height-difference threshold *th_h-diff_* (**left**) and error comparison with and without merging (**right**).

As to text-region merging, we performed similar tests in order to analyze the effect of moving the two thresholds (horizontal distance *th_h-dist_* and height difference *th_h-diff_*) and evaluating the overall effectiveness of the approach. Being that the two thresholds are practically independent, we first evaluated the effect of moving the first with the second one set at a default value, then we moved the second one with the first set at the value suggested by the first test. Figure 9 and Figure 10 (left part) show that we a have good trade offs at *th_h-dist_* = 0.1 and *th_h-diff_* = 0.5. The evaluation of the effectiveness of the merging approach (with the above threshold values) on the main dataset is shown in the right part of Figure 10: the two metrics are basically confirmed at 2% and 0.1% for our approach (as opposed to 15% and 2.54%, respectively, when no box merging is performed).

**Effectiveness of OCR/text extraction**. We will now discuss the results of the evaluation of the OCR libraries, as described in Section 3.1, and the effectiveness that our tool is able to reach. The metrics *qdiff* and *qscore* (as defined in Section 3.1) were used. In particular, the input quality oq was defined in a range from 0 (a low-quality scan of a page that contains a lot of noise, or poorly defined or damaged writing) to 2 (a well-defined, high-quality scan); as to output quality, it was evaluated by linguistic experts on a range from 0 (completely wrong results) to 2 (completely correct results).

**Figure 11:**
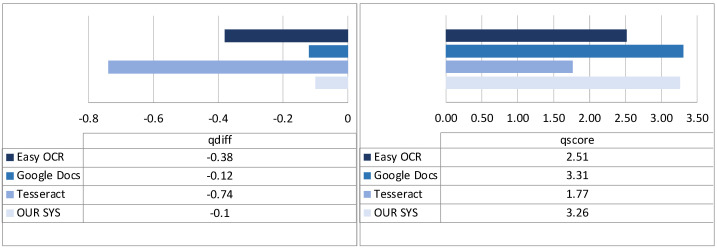
Text extraction: qdiff (**left**) and qscore (**right**) overall results.

Figure 11 shows the average performance of each system in terms of *qdiff* (left part of figure) and *qscore* (right part of figure). Starting our analysis from state-of-the-art systems, as we can see from the *qdiff* metric, GoogleDocs generally performs better than EasyOCR (with a score near 0, confirming an output that is typically in line with the quality of the processed input), while the worst-performing library is Tesseract, with a *qdiff* near −1. This is also confirmed by the *qscore* values. As to our approach, we can see that its scores are slightly better than GoogleDocs, which is the best-performing system. In particular, this is the first evidence that the best output selection strategy, based on the count of words found in existing multilingual corpora, is working well (indeed, in this regard, we verified that, on the sample of documents considered, our approach correctly classifies the best output in 95% of cases).

**Figure 12:**
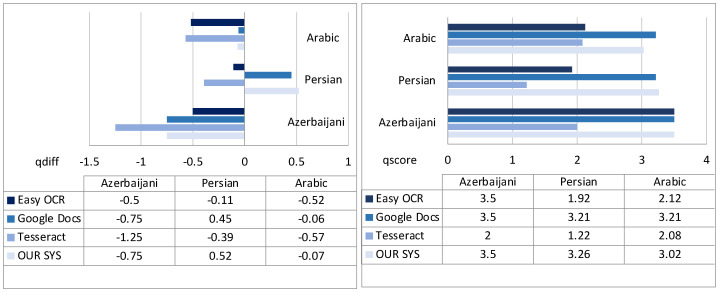
Text extraction: qdiff (**left**) and qscore (**right**) results per language.

Moreover, we were also interested in analyzing the comparative performances w.r.t. the specific languages of the sample documents (results shown in Figure 12). As we can see, considering state-of-the-art libraries and *qdiff* (left part of figure), we see that some languages are more difficult to deal with than others (e.g., Azerbaijani), while only for Persian do some systems provide an output quality exceeding the input quality of the documents (positive *qdiff* scores). In particular, the best-performing system for Azerbaijani is EasyOCR, with GoogleDocs being very close (also looking at *qscore* on the right), then Tesseract.

GoogleDocs appears as the general best choice (in particular for Persian and Arabic, with good results also for Azerbaijani); anyway, by going beyond the average values shown in the graphs and analyzing the performance on the single document cases, we note that there are indeed some cases (especially for Arabic and Persian) where GoogleDocs is not always able to return a better output than others.

**Figure 13:**
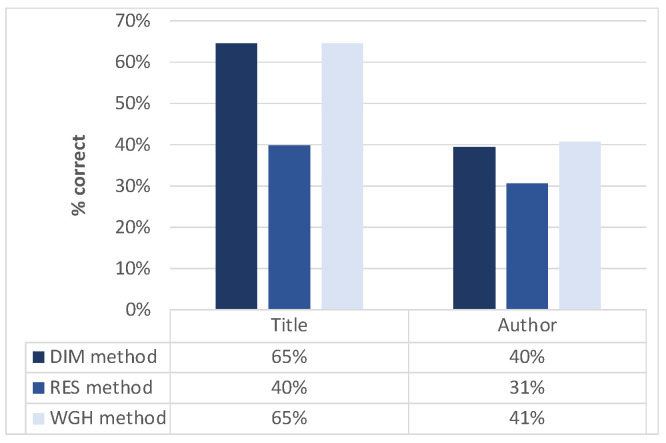
Title/author identification: correctness of the best guess for the different methods.

**Effectiveness of title/author identification**. Figure 13 shows the accuracy (% of correct guesses) we currently achieve on the considered dataset for the three methods discussed in Section 3.1. The DIM and WGH method achieve the best accuracy for both title (65% for both) and author (40% and 41%, respectively) and their performance is quite close. We have to remember that the quality of the scanned images is generally quite low (this is to reflect the actual digital data that is available to cataloguers) and this, in some cases, prevents the full exploitation of the external linguistic resources’ potential on the extracted text. While the overall figures can certainly be improved, in any case, they represent a promising result since none of the systems available in the state of the art projects aims at automating this task, thus requiring the completely manual insertion/selection of both titles and authors. In the future, we will consider further improvements to the methods so that the system will adapt to the quality of the documents and only provide the suggestions for which it is most confident.

### 4.3. Future Work

Generally speaking, besides the specific improvements to the presented methods discussed in the relevant sections, many are the steps we envision that will lead to the creation of the final complete supervised intelligent cataloguing tool, for which we will also exploit our past expertise in semantic [32] and machine-learning techniques in different scenarios [33,34,35]:

- From a data-management perspective, special focus will be given to the data interchange and long-term preservation aspects, in order to allow data interchange with catalogue data from other libraries and make the managed data readable and usable also on a long-term basis [36];
- Intelligent and AI-based techniques will also have a prominent role: intelligent assistance features will be designed and implemented in order to bring new levels of assistance to the cataloguing process. Data entering will be supported by suggestions derived from user feedback and previously entered data, thus integrating and extending the author-/title-identification techniques we described in this paper; supervised machine-learning models will enable automatic publication-type recognition and provide a systematisation and classification of data according to the topographic design of the La Pira library;
- The design of incremental ML algorithms will ensure that the tool can “learn” and become more and more automated and effective with use. Both classic and deep-learning algorithms will be considered, deployed on parallel architectures for faster execution. Special attention will be given to interpretable machine-learning algorithms, following the recent interpretable machine-learning trend in different fields [37], with the aim of going beyond the black-box nature of ML suggestions and explaining them, also in the library cataloguing/cultural heritage context, where this has seldom been performed;
- Ee plan to extend the scope of the experimental tests by considering incrementally larger portions of the use case library.

Al techniques will be integrated in order to create a reproducible and reusable web tool enabling a simple cataloguing workflow overcoming language and field obstacles.

## 5. Conclusions

In this paper, we presented the first steps for designing a novel tool for the automatic extraction/sensing of knowledge from documents written in multiple non-Latin languages. As shown in the preliminary tests, the performance of the currently developed techniques are encouraging. We will conclude the paper by briefly discussing the expected advantages of the final tool we are aiming to create, both from technical and non-technical perspectives.

From a technical point of view, many foreseen advantages are auspicable in the scientific domain:

- *Overcoming the limitations of current text-extraction tools*: non-uniform OCR with different characters, backwardness of OCR for Arabic, poor automation and extraction of additional metadata;
- *Faster pipeline*: To date, the cataloguing of documents has been performed manually, document by document, opening them one at a time, searching inside the information to be catalogued and then moving to the next one. By employing the tool being developed, we can drastically reduce the time spent cataloguing simpler documents (since the suggested output will need fewer modifications from the user), while for the most complex ones, the system can still provide useful suggestions. In both cases, this system will speed-up the entire procedure;
- *Greater consistency and fewer errors*: In fact, with the automatic suggestions supplied to the user, it will become easier to avoid mistakes while inserting the document’s information, making the extracted data less error-prone. Moreover, the system will constantly perform checks on previously catalogued data in order to avoid inconsistencies, which can be very common in manual cataloguing (e.g., an author surname inserted with/without name, abbreviated name, etc.);
- *Consistently better system output through time*: by means of the machine-learning/intelligent features, the system will provide an output that will be better and better as the system is used, exploiting its “training” on previously catalogued, similar documents;
- *Flexibility of data output/exchange*: being a system based on a complete data-management system on which all the user and system output is stored, the system can at any time access the stored data and convert the bibliographic information toward most desired outputs, thus facilitating data exchange;
- *Efficiency and Explainability*: advanced data-management and machine-learning techniques will enable high efficiency levels for significant data-cataloguing needs, while the explainability of the models will make the intelligent assistance tools more usable.

Finally, from a broader standpoint, benefits from this research and the use of the tool are expected on several innovative fronts:

- Advancement of studies on cataloguing in multi-literate environments without leaning exclusively on confusing transliteration systems;
- Exchange of IT, humanist and library personnel, enhancement of professional skills, training activities extended to realities with similar needs;
- Strengthening of library services thanks to shared international standards, expanding library heritage, databases integration, maximum access to the heritage, possibility of using the language of the document without the mediation of other languages.

We are also aware of some limitations of our current research: in particular, achieving complete automation might be, in some ways, limited by the quality of the input images, which is not always satisfying; moreover, we are aware that text sensing on Arabic-script documents is an area where available research results and tools are not as developed as the ones for Latin scripts. This poses some obstacles but it is also a further motivation for what we are performing. Finally, machine-learning/intelligent features will only be possible with large amounts of data already loaded into the system, which will require a significant initial amount of manual work. In any case, we are confident that these limitations will be dealt with in our future research work.

In short, we are confident that this research will ultimately help in preserving and conserving culture, a crucial task, especially in the challenging scenario we consider.

## Data Availability

At present the dataset of the La Pira Library is not publicly available, all items were donated from different contributors to the La Pira Library in PDF format and are property of the Fondazione per le Scienze Religiose (FSCIRE) and “Giorgio La Pira” Library. It is worth to remind that the main goal of the present research is to enable the creation of a digital library that will eventually enable to make those big amounts of data available, thus providing access to a wide array of users, from students and researchers to the general public.

## Abbreviations

*oq*	manually assessed OCR output quality
*iq*	manually assessed OCR input quality
*qdiff*	resulting quality difference from *oq*-*iq*
*qscore*	overall quality of the OCR system result, given the input quality
*v-dist*	vertical distance
*h-dist*	horizontal distance
*h-diff*	text boxes height difference
*th_v-dist_*	Threshold on vertical distance
*th_h-diff_*	Threshold on height difference
*th_h-dist_*	Threshold oh horizontal distance
*h*	boxes height
*g*	box(es) group
